# Long COVID and its associations with burnout, anxiety, and depression among U. S. healthcare workers in the United States

**DOI:** 10.3389/fpubh.2025.1582872

**Published:** 2025-07-09

**Authors:** Thanh-Huyen T. Vu, Miao Jenny Hua, Cerina Dubois, Judith T. Moskowitz, Amisha Wallia, Lisa R. Hirschhorn, John T. Wilkins, Charlesnika T. Evans

**Affiliations:** ^1^Department of Preventive Medicine, Feinberg School of Medicine, Northwestern University, Chicago, IL, United States; ^2^Chicago Department of Public Health, Chicago, IL, United States; ^3^Department of Mental Health, Bloomberg School of Public Health, Johns Hopkins University, Baltimore, MD, United States; ^4^Department of Medical Social Sciences, Feinberg School of Medicine, Northwestern University, Chicago, IL, United States; ^5^Institute for Public Health and Medicine, Center for Health Services and Outcomes Research, Northwestern University, Chicago, IL, United States; ^6^Department of Medicine, Division of Endocrinology, Metabolism, and Molecular Medicine, Feinberg School of Medicine, Northwestern University, Chicago, IL, United States; ^7^Robert J. Harvey Institute for Global Health, Northwestern University, Chicago, IL, United States; ^8^Department of Medicine, Division of Cardiology, Feinberg School of Medicine, Northwestern University, Chicago, IL, United States; ^9^Department of Veterans Affairs, Center of Innovation for Complex Chronic Healthcare, Edward Hines, Jr. VA Hospital, Hines, IL, United States

**Keywords:** healthcare workers, long COVID, burnout, anxiety, depression, mental health

## Abstract

**Background:**

Data on Long COVID and its associations with burnout, anxiety and depression among healthcare workers (HCW) in the United States (U. S.) is limited.

**Methods:**

This study utilized cross-sectional data from the final survey conducted in July 2023, which was part of a longitudinal cohort study assessing COVID-19-related burnout and wellbeing among healthcare workers (HCWs) in a large tertiary academic healthcare system in the Chicago area. The survey included questions on self-reported Long COVID status, as well as the Oldenburg Burnout Inventory (OLBI) to measure burnout and the Patient-Reported Outcomes Measurement Information System (PROMIS) computer adaptive tests (CAT) to assess anxiety and depression. A total of 1,979 HCWs participated in the survey, yielding a response rate of 56.1%.

**Results:**

The analysis included 1,678 respondents with complete data, of whom 1,171 (70%) self-reported having had COVID-19. Of these, 90 (7.7%) reported Long COVID, with 53% indicating that their most bothersome symptoms persisted for more than 6 months, while 50% reported no longer experiencing those symptoms at the time of the survey. Multivariable linear regression analyses revealed that Long COVID was significantly associated with higher OLBI scores (*β* = 2.20, *p* = 0.004), PROMIS anxiety scores (*β* = 2.64, *p* = 0.001) and PROMIS depression scores (*β* = 1.98, *p* = 0.011) compared to those who had COVID-19 but not Long COVID. Similar patterns of associations were observed when comparing the Long COVID group to those who never had COVID-19. No significant differences were found between those who never had COVID-19 and those who had COVID-19 without developing Long COVID.

**Conclusion:**

Long COVID was associated with higher levels of burnout, depression, and anxiety among healthcare workers compared to those who had COVID-19 alone or were never infected, despite its lower prevalence during the endemic phase. These findings underscore the need for continued prevention efforts and targeted support strategies in healthcare settings.

## Introduction

Long COVID, also known as post-COVID-19 syndrome or post-acute sequelae of COVID-19, refers to the wide range of signs, symptoms, and conditions that can persist for weeks, months, or even years following an initial SARS-CoV-2 infection (1–4). Estimates of the prevalence of Long COVID have varied widely, ranging from <10% (5) to over 80% (6, 7), depending on factors such as the population studied, the timing of assessment, and the diagnostic criteria used. In the general population, Long COVID has been implicated in up to 15% of unfilled jobs (8) and is associated with elevated levels of fatigue and mental health disorders (9, 10) highlighting its significant impact on workforce participation and functional capacity (11).

Despite this, comprehensive data on the associations between Long COVID, burnout, and mental health outcomes in healthcare workers (HCWs)—a critical workforce—remain limited, particularly in the United States (U. S.). HCWs have faced disproportionate exposure to SARS-CoV-2 infection (12) and exhibited the highest prevalence of Long COVID among occupational groups during the pandemic (13). Those affected report significantly lower quality of life and higher rates of depression and anxiety compared to workers in other sectors (14). However, most existing studies were (12, 15–23) conducted outside the U. S., relied primarily on descriptive analyses (14–19), or involved small sample sizes (14). Moreover, most were carried out during the pandemic phase, spanning early 2020 to the end of 2022.

As COVID-19 has transitioned to an endemic phase, the prevalence of Long COVID in the U. S. has declined and appears to have stabilized—likely due to widespread vaccination and increased population-level immunity (20). Nonetheless, the mental health consequences of Long COVID may continue to pose a significant public health challenge, particularly for HCWs. It is therefore critical to assess and contextualize the ongoing burden of Long COVID during this endemic phase to inform targeted interventions and guide future research.

In this study, using cross-sectional data from the July–August 2023 survey of the Northwestern Medicine HCW Cohort Study, we aimed to (1) describe HCWs’ self-reported history of Long COVID since the onset of the COVID-19 pandemic in early 2020, and (2) examine its associations with burnout, anxiety, and depression levels assessed in mid-2023, accounting for other related factors. To our knowledge, this is the first large-scale study in the U. S. to explore these associations among HCWs across both the pandemic and endemic phases of COVID-19 (21). We also aimed to assess whether these associations (if any) were driven by Long COVID, COVID-19 alone, or to both conditions.

## Methods

Reporting for this study followed the EQUATOR Strengthening the Reporting of Observational Studies in Epidemiology (STROBE) guidelines (Appendix 1). This study was approved by the Northwestern University Institutional Review Board (STU00212515) prior to recruitment of HCWs, and all participants provided written informed consent at study enrollment.

### Study design and population

The study is a cross-sectional survey within a longitudinal cohort study. Between May 28 and June 30, 2020, a cohort of 6,510 Northwestern Medicine HCWs were enrolled in the Northwestern Medicine Healthcare Worker SARS-CoV-2 Serology Cohort Study. Details of the study designs, as well as serology and survey results, have been published previously (22, 23). In June 2021, the study was extended, with 3,569 HCWs consenting to participate in ongoing serial surveys that included questions on COVID-19, burnout and psychological wellbeing. The final survey, conducted between July 10 and August 21, 2023, was sent to 3,530 participants (after excluding those who withdrew or were lost to follow-up), and 1,979 responded (response rate: 56.1%). This iteration included new questions regarding Long COVID status. For the analysis, we excluded 226 respondents who did not complete the Oldenburg Burnout Inventory (OLBI) or Patient-Reported Outcomes Measurement Information System (PROMIS) anxiety and depression assessment, along with 54 individuals missing key covariate data. Additionally, 21 individuals who reported testing positive for COVID-19 within the previous month were excluded from the main analysis, as insufficient time may have passed for Long COVID to develop. These exclusions resulted in a final analytic sample of 1,678 participants (Supplementary Figure 1).

### COVID-19 and long COVID ascertainment

Participants were asked to self-report whether they ever had COVID-19, with response options of “yes,” “no,” or “unsure.” They were also asked if they had experienced Long COVID, defined as one or more “symptoms of COVID-19 4 weeks or later after being infected or suspecting to have been infected with COVID-19,” which “can sometimes appear after recovering from the initial infection.” The response options for Long COVID were: “yes, diagnosed by a health professional,” “yes, self-diagnosed,” “unsure,” and “no.” This definition aligns with the U. S. federal working definition of Long COVID (2), as used in U. S. national surveys (24), before the consensus definition was launched in 2024 (25). Common post-COVID symptoms listed included general, respiratory and cardiac, neurological, digestive, and other symptoms, such as fever, weak or tired, shortness of breath, cough, chest pain, loss of smell or taste, nausea, vomiting, etc. (24).

Initially, “unsure” responses for COVID-19 were combined with “yes,” as they may represent individuals who experienced symptoms but did not undergo testing. Participants were categorized into four groups based on their Long COVID status: (1) had COVID-19 with Long COVID (COVID+/LC+); (2) had COVID-19 without Long COVID (COVID+/LC-); (3) had COVID-19 but unsure about Long COVID (COVID+/LC?); and (4) never had COVID-19 and therefore no Long COVID (COVID-/LC-). Subsequently, we excluded participants who were “unsure” about either having COVID-19 or Long COVID, refining the categorization of Long COVID status into three groups: COVID+/LC-, COVID+/LC+, and COVID-/LC-to focus on confirmed responses.

Participants who reported having Long COVID were further asked questions regarding the presence of 19 specific symptoms, symptom duration, functional impairment, recent COVID-19 testing (within the past month), and their current Long COVID status.

### Burnout, anxiety, and depression

Burnout was assessed using the OLBI, a 16-item inventory which measures exhaustion and disengagement from work, which are core components of burnout (26). The OLBI has been validated among English-speaking workers in the United States. Responses range from 1 (totally disagree) to 4 (totally agree), with total scores ranging from 16 to 64 (27). Individuals with an average OLBI disengagement score of 2.1 or greater and an average OLBI exhaustion score of 2.25 or greater were classified as having burnout (28).

Anxiety and depression were measured using items of the PROMIS computer adaptive tests (CAT) (29). These psychological measures were reported as T-scores (M = 50, SD = 10) of the general population. For both PROMIS depression and anxiety assessments, a T-score of 55 or below is considered normal, while scores above 55 indicate the presence of anxiety or depression (30, 31).

### Covariates

Age, number of COVID-19 vaccinations (including boosters), and comorbidity status were self-reported at the current survey. Sex, race/ethnicity, occupation, height, weight, and occupation were self-reported at baseline in the Northwestern Medicine Healthcare Worker SARS-CoV-2 Serology Study (22). Race/ethnicity was categorized as Hispanic, non-Hispanic White, Asian, and Other groups. Occupations were classified into four major groups: administration, nurses, physicians, and other roles (e.g., clinical/education coordinator, dialysis technician, environmental service). Body mass index (BMI) was calculated from self-reported height and weight. The number of comorbidities was based on a list of 13 chronic diseases: asthma requiring medication, cancer, chronic kidney disease, coronary heart disease [heart attack, stent, or coronary artery bypass grafting (CABG)], diabetes mellitus, dialysis for kidney failure, emphysema/ chronic obstructive pulmonary disease (COPD), heart failure, high blood pressure, immunocompromised state, liver disease, obesity, and other chronic lung diseases.

### Statistical analysis

Descriptive analysis was conducted to assess the prevalence of Long COVID and to describe the sociodemographic and clinical characteristics of the participants. F-tests and chi-square tests were used to compare differences in participant characteristics across the four Long COVID status groups. Additionally, respondents to the current survey (*n* = 1979) were compared to non-respondents (*n* = 1,551) to determine whether there were any significant differences in their socio-demographic and comorbidity characteristics.

For the main analysis, multivariable linear regression analysis was used to examine the associations between Long COVID status and OLBI burnout scores, as well as PROMIS anxiety, and PROMIS depression scores. Models were adjusted for age, sex, and race/ethnicity, occupation, BMI, comorbidity, and the number of COVID-19 vaccinations. Pairwise comparisons were conducted to identify significant differences among the four groups.

In exploratory analyses, multivariable logistical regression was performed with burnout, anxiety, and depression as dichotomous outcomes.

For the sensitivity analyses, first, we excluded 209 individuals who were “unsure” about their COVID-19 or Long COVID status. As a result, the analyses were conducted among only participants with confirmed responses for both COVID-19 and Long COVID status (*n* = 1,469). Second, to assess potential bias due to nonresponse, we applied inverse probability weighting based on demographic and occupational characteristics that significantly differed between responders and non-responders (see Supplementary Table 3).

All analyses were performed using SAS (SAS Institute Inc., Cary, NC, USA), with statistical significance set at *p* < 0.05.

## Results

**Figure 1:**
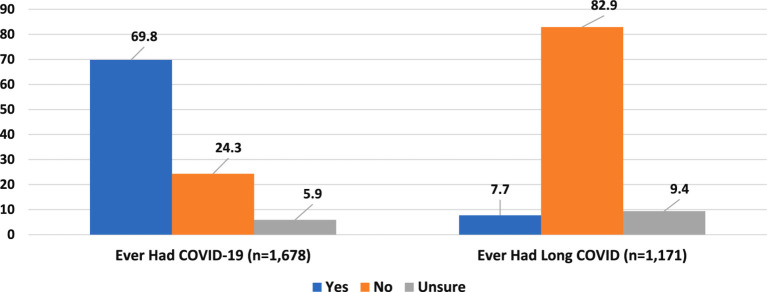
Self-reported COVID-19 and long COVID prevalence in analysis sample.

Figure 1 shows the prevalence of COVID-19 and Long COVID in the analysis sample. Among the 1,678 HCWs, 1,171 (70%) reported having had COVID-19. Of these, 90 (7.7%) reported experiencing Long COVID, while 110 (9.4%) were unsure about their Long COVID status.

Table 1 presents the characteristics of participants, overall and by Long COVID status. The mean age of the analysis sample was 48.1 years (SD = 11.6), with the majority being female (81.8%) and non-Hispanic White people (81.7%). Nurses represented the largest occupational group (28.4%) aside from the composite “other” category. Over half (56.7%) reported having no comorbidities, and most had received 3 or more doses of the SARS-CoV-2 vaccine (83.8%).

**Table 1:** Sociodemographic and clinical characteristics of survey respondents (*N* = 1,678).

Characteristic	Overall *N* = 1,678	Long COVID status				
		COVID+/LC+ *N* = 90	COVID+/LC- *N* = 1,057	COVID+/LC? *N* = 123	COVID-/LC- *N* = 408	*p*-value ^c^
Age, mean (SD)	48.1 (11.6)	46.1 (10.8)	47.2 (11.4)	48.6 (11.1)	50.8 (11.8)	<0.0001
Female [*n*(%)]	1372 (81.8%)	71 (78.9%)	862 (81.6%)	98 (79.7%)	341 (83.6%)	0.617
Race/ethnicity group [*n*(%)]						
Hispanic	103 (6.1%)	12 (13.3%)	62 (5.9%)	12 (9.8%)	17 (4.2%)	0.046
Asian	142 (8.5%)	7 (7.8%)	94 (8.9%)	12 (9.8%)	29 (7.1%)	
Non-Hispanic White	1371 (81.7%)	69 (76.7%)	865 (81.8%)	94 (76.4%)	343 (84.1%)	
Other	62 (3.7%)	2 (2.2%)	36 (3.4%)	5 (4.1%)	19 (4.7%)	
Occupation category [*n*(%)]						
Administration	232 (13.8%)	16 (17.8%)	141 (13.3%)	12 (9.8%)	63 (15.4%)	0.001
Nurse	476 (28.4%)	22 (24.4%)	305 (28.9%)	35 (28.5%)	114 (27.9%)	
Physician	314 (18.7%)	14 (15.6%)	221 (20.9%)	8 (6.5%)	71 (17.4%)	
Others	656 (39.1%)	38 (42.2%)	390 (36.9%)	68 (55.3%)	160 (39.2%)	
Number comorbidities^a^ [*n*(%)]						
0	952 (56.7%)	50 (55.6%)	639 (60.5%)	56 (45.5%)	207 (50.7%)	0.002
1	490 (29.2%)	28 (31.1%)	294 (27.8%)	38 (30.9%)	130 (31.9%)	
2	158 (9.4%)	8 (8.9%)	86 (8.1%)	18 (14.6%)	46 (11.3%)	
3 or more	78 (4.6%)	4 (4.4%)	38 (3.6%)	11 (8.9%)	25 (6.1%)	
Number of vaccinations^b^ [*n*(%)]						
<3 shots	272 (16.2%)	23 (25.6%)	166 (15.7%)	29 (23.6%)	54 (13.2%)	<0.0001
3 shots	587 (35.0%)	34 (37.8%)	392 (37.1%)	46 (37.4%)	115 (28.2%)	
4+	819 (48.8%)	33 (36.7%)	499 (47.2%)	48 (39.0%)	239 (58.6%)	
BMI (kg/m^2^), mean (SD)	27.7 (6.3)	28.2 (6.0)	27.3 (6.0)	29.7 (6.9)	28.0 (6.6)	0.0003

COVID+/LC+, had COVID-19 with Long COVID; COVID+/LC-, had COVID-19 without Long COVID; COVID+/LC?, had COVID-19 but unsure about Long COVID; COVID-/LC-, never had COVID-19; BMI: Body mass index. ^a^Derived from a list of 13 chronic diseases. ^b^Number of COVID-19 vaccinations including boosters. ^c^*P*-value for overall group comparisons based on F-tests or Chi-square tests.

Age, race/ethnicity, occupation, BMI, number of comorbidities and vaccinations significantly varied by Long COVID status. The COVID+/LC + group tended to be younger (mean age = 46.1), had a lower proportion of non-Hispanic White people (76.7%), a higher proportion in administrative roles (17.8%), and a lower rate of receiving three or more vaccine doses (74.5%) compared to other groups, particularly the COVID+/LC-and COVID-/LC-groups (*p* < 0.05).

**Figure 2:**
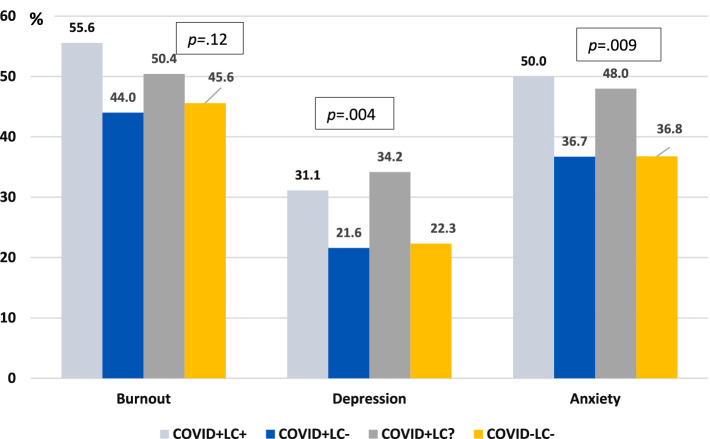
Prevalence of burnout, depression, and anxiety by prior COVID-19 and long COVID status. COVID+/LC+, had COVID-19 with Long COVID; COVID+/LC-, had COVID-19 without Long COVID; COVID+/LC?, had COVID-19 but unsure about Long COVID; COVID-/LC-, never had COVID-19; Burnout was defined as having both OLBI-Exhaustion score ≥ 2.25 and OLBI-Disengagement score ≥ 2.1; Depression and anxiety defined as PROMIS T-scores ≥55. *p*: *p*-value for overall group comparisons based on Chi-square tests.

In the overall sample, 45.5% of participants met the criteria for burnout, 38.3% for anxiety, and 23.2% for depression. Significant differences were observed by Long COVID status in prevalence of anxiety (*p* = 0.009) and depression (*p* = 0.004) (Figure 2).

In multivariable linear regression analyses with adjustment for age, sex, occupational status, BMI, number of comorbidities and number of vaccines received, self-reported Long COVID (COVID+/LC+) was significantly associated with higher OLBI scores (*β* = 2.20, SE = 0.77, *p* = 0.004), PROMIS depression scores (*β* = 1.98, SE = 0.78, *p* = 0.011), and PROMIS anxiety scores (*β* = 2.64, SE = 0.77, *p* = 0.001) compared to the COVID+/LC- group. Similar associations were observed when comparing the COVID+/LC + group with the COVID-/LC- group. Those in the COVID+/LC? Also had higher PROMIS depression and anxiety scores compared to the COVID+/LC- group. No significant differences in burnout, anxiety, or depression scores were observed between the COVID+/LC- group and the COVID-/LC- group (Table 2).

**Table 2:** Multivariable linear regression analysis of the association between long COVID and OLBI and PROMIS depression and anxiety scores (*N* = 1,678).

Long COVID status comparisons	OLBI *β* (SE)	*p*-value	PROMIS depression *β* (SE)	*p*-value	PROMIS anxiety *β* (SE)	*p*-value
COVID+/LC + vs COVID+/LC-	2.20 (0.77)	0.004	1.98 (0.78)	0.011	2.64 (0.77)	0.001
COVID+/LC? vs COVID+/LC-	0.94 (0.68)	0.166	1.68 (0.68)	0.014	2.04 (0.68)	0.003
COVID+/LC + vs COVID+/LC?	1.26 (0.97)	0.195	0.30 (0.98)	0.759	0.61 (0.97)	0.534
COVID+/LC + vs COVID-/LC-	1.73 (0.82)	0.035	1.53 (0.83)	0.065	2.41 (0.82)	0.004
COVID+/LC- vs COVID-/LC-	−0.47 (0.41)	0.259	−0.45 (0.42)	0.277	−0.23 (0.41)	0.570
COVID+/LC? vs COVID-/LC-	0.47 (0.73)	0.519	1.23 (0.73)	0.094	1.80 (0.73)	0.014

COVID+/LC+, had COVID-19 with Long COVID; COVID+/LC-, had COVID-19 without Long COVID; COVID+/LC?, had COVID-19 but unsure about Long COVID; COVID-/LC-, never had COVID-19; OLBI: Oldenburg Burnout Inventory; PROMIS: Patient-Reported Outcomes Measurement Information System. Models adjusted for age, sex, occupational status, BMI, number of comorbidities and number of vaccines received.

The adjusted mean OLBI, PROMIS Depression, and Anxiety Scores by Long COVID Status are presented in Supplementary Figure 2, which aligns with the findings in Table 2. For example, no significant difference in OLBI scores between the COVID+/LC + and COVID+/LC? groups (34.7 and 35.6, *p* = 0.166), or between the COVID+/LC-and COVID-/LC-groups (34.7 and 35.1, *p* = 0.259). However, significant differences were observed between OLBI scores of the COVID+/LC + and COVID+/LC-groups (36.9 and 34.7, *p* = 0.004), as well between the COVID+/LC-and COVID-/LC-groups (36.9 and 35.1, *p* = 0.035).

In the sensitivity analyses, excluding 209 participants who responded “unsure” to questions about COVID-19 and Long COVID status, similar associations between Long COVID status and OLBI, PROMIS depression and PROMIS anxiety scores were observed, consistent with the main analysis (Supplementary Table 1). Applying inverse probability weighting to account for differences between responders and non-responders slightly reduced the effect sizes, but the results remained significant, consistent with the primary findings (results not shown).

In the exploratory analyses, the odds of experiencing burnout, depression, and anxiety were higher in the COVID+/LC + group compared to the COVID+/LC- group by 69, 65, and 78%, respectively, although the association with depression was not statistically significant. No significant differences in the odds of burnout, anxiety, or depression were observed between the COVID+/LC- group and the COVID-/LC- group (Supplementary Table 2). Among the 90 participants in the COVID+/LC + group, the top three most bothersome symptoms reported were weakness/fatigue (36.7%), loss of taste or smell (34.4%), and headache (17.8%). Additionally, 53.3% (48/90) of participants reported experiencing their most bothersome Long COVID symptoms for more than 6 months. Notably, half of participants with Long COVID (45/90) reported no longer experiencing their most bothersome Long COVID symptoms (results not tabulated).

## Discussion

By July–August 2023, more than two-thirds of healthcare workers surveyed in this cohort self-reported having had COVID-19; among them, 7.7% reported Long COVID. Over half of those with Long COVID indicated their most bothersome symptoms persisted for more than 6 months. The prevalence of Long COVID in our cohort (7.7% of those reporting ever had COVID-19) was notably lower than previous reports among HCWs. Even when including those who were “unsure” about having Long COVID, the prevalence rises only to 17.1%. In contrast, earlier studies of HCWs, primarily from North America and Western Europe, have reported Long COVID prevalence ranging from 26% to over 40% (15, 16, 32, 33). A systematic review found a median prevalence of 47.7% across 19 studies of HCWs (34). Similarly, a U. S. study reported that 60.6% of vaccinated and 79.1% of unvaccinated HCWs experienced Long COVID (18). However, these higher estimates were largely calculated earlier in the pandemic when vaccination rates were low, and the original SARS-CoV-2 strain was more prevalent (15, 16, 18, 32).

Our findings are more aligned with a study in Saudi Arabia, which reported a 15.3% prevalence of self-reported Long COVID (symptoms persisted > 1 month) among vaccinated HCWs infected between May and August 2022 (35). In our cohort, 99% of HCWs had received at least one dose of a COVID-19 vaccine, with 84% having received three or more doses, suggesting that most had either vaccine-induced or hybrid immunity. Moreover, our prevalence was derived across both the pandemic and endemic phases of COVID-19 transmission, which may contribute to the relatively lower rate of Long COVID.

It is important to note that the consensus definition of Long COVID has evolved recently, specifying that Long COVID is a condition persisting for at least 3 months after infection with SARS-CoV-2 (25). Before this, studies varied in their criteria for Long COVID, making direct comparisons across findings difficult. Additionally, the timing of data collection plays a significant role, as vaccination rates, viral variants, and the changing nature of the pandemic likely influenced Long COVID prevalence over time.

After adjusting for covariates, healthcare workers with Long COVID in our study had significantly higher levels of burnout, anxiety, and depression compared to those who had COVID-19 but did not have Long COVID as well as those who never had COVID-19. However, no significant differences in these outcomes were observed between individuals who had COVID-19 without Long COVID and those who never had COVID-19, suggesting that burnout and poor mental health among healthcare workers may be more strongly associated with Long COVID than with COVID-19 experience alone. Long COVID has been associated with mental health issues such as depression, anxiety (9), and fatigue in general population (36). However, comprehensive data on these associations in healthcare settings, particularly in the U. S., remain limited. Our findings align with a cross-sectional study of HCWs in Boston, Massachusetts, conducted between September 2022 and January 2023, which used a similar definition of Long COVID and a validated mental health instrument (DASS-21). That study found that HCWs with Long COVID had higher levels of depression, anxiety, and poorer quality of life (14). However, it lacked a measure of burnout as well as individuals without COVID-19 as the reference group, limiting its ability to determine whether burnout and mental health outcomes was driven by Long COVID specifically. It also used a smaller sample (*n* = 280) and was conducted earlier than our study, without data from the endemic phase.

Our findings are also consistent with a national survey of the UK nephrology workforce (37), which found that individuals with Long COVID were 10 times more likely to experience burnout than those without. This association is notably stronger than what we observed, potentially due to differences in the timeframe and the definition of Long COVID used. As in our study, no association was found between individuals who had COVID-19 without developing Long COVID and those who were never infected (37).

A study of 1,490 primary care professionals of Spain found that both those with acute COVID-19 and those with Long COVID had a higher prevalence of anxiety compared to those without COVID-19, with stronger associations seen for Long COVID. However, that study did not account for confounding factors, limiting the comparability of its findings (38). In our study, we also found that HCWs who were unsure if they had Long COVID reported higher levels of mental distress than those who had COVID-19 without Long COVID. This uncertainty about a Long COVID diagnosis may itself contribute to heightened anxiety (39).

Notably, our survey was conducted over 3 years after the start of the COVID-19 pandemic, and half of respondents with Long COVID reported no longer experiencing their most bothersome symptoms. Despite this, we still observed elevated levels of burnout, anxiety and depression among those with Long COVID. These findings suggest that Long COVID, rather than prior COVID-19 experience alone, may continue to affect burnout and mental health among HCWs, even after a significant period of recovery.

While fatigue, depression, and anxiety are established Long COVID symptoms (36), we analyzed them as distinct mental health outcomes using validated instruments to better capture the broader psychological burden of the condition. Our goal was not to infer causality but to examine these association in a real-world occupational context, accounting for relevant factors such as demographics, job type, vaccination status, and comorbidities.

Key strengths of our study include its large-scale, comprehensive assessment of Long COVID’s associations with burnout, depression, and anxiety among U. S. HCWs. The inclusion of a cross-product between Long COVID and COVID-19 status enabled a more nuanced evaluation of group differences. To our knowledge, this is the first study to span both the pandemic and endemic phases of COVID-19, offering updated prevalence of Long COVID and insights into its associations with burnout and mental wellbeing among U. S. HCWs. Although Long COVID prevalence has declined, recent evidence suggests it has plateaued rather than significantly decreased (40). Given the profound impact of burnout and mental health issues among HCWs—affecting not only individual wellbeing but also patient care and overall system performance (41)—our findings have important real-world implication. They support the need for continued prevention efforts and underscore the importance of interventions that address the mental health consequences of Long COVID in HCWs. Specific workplace policies, such as regular mental health screenings and flexible sick leave provisions, are crucial for addressing these ongoing challenges.

However, our study has limitations. First, it relied on a convenience sample from a single healthcare system in Chicago and surrounding suburban counties, with a majority of participants identified as White and female, and notable non-response rate. There were also statistically significant differences between responders and non-responders in terms of age, race, occupational status, and comorbidities (Supplementary Table 3), which may limit the generalizability of our findings to the broader HCW population. However, the sensitivity analysis applying inverse probability weighting yielded results consistent with the main findings, reinforcing our conclusions.

Second, mental health outcomes among healthcare workers are influenced by a range of factors beyond COVID-19 or Long COVID, including occupational role, work environment, decision-making autonomy, and personal circumstances such as family responsibilities. Our dataset does not capture all of these variables. However, we addressed some of this complexity by including occupation types (i.e., nurses, doctors, or administrators) in our multivariable regression models and we still observed significant associations between Long COVID and mental health outcomes.

Third, although the cohort was prospectively established, the lack of pre-pandemic data and the cross-sectional nature of this analysis limits our capacity to assess longitudinal changes in Long COVID or mental health outcomes. Studies in general populations suggest a bidirectional association between COVID-19 and psychiatric disorder (42). However, Long COVID has been associated with an increased risk of developing mental health conditions such as depression and anxiety (9). Recent findings indicate that patients with persistent Long COVID symptoms have low serotonin levels, potentially implicating a pathophysiological link between Long COVID, serotonin dysregulation, and depression (43). This underscores the need for future research to elucidate the causal relationship and underlying mechanisms connecting Long COVID with burnout and mental health.

Furthermore, our self-reported data on Long COVID and mental wellbeing shares a common information bias with existing studies on this topic. However, any possible misclassification would be similar across comparison groups (non-differential), i.e., it would underestimate the association. Nevertheless, we still found different associations across group comparisons.

## Conclusion

The prevalence of Long COVID among U. S. healthcare workers in this study, conducted 3 years after the onset of the COVID-19 pandemic, was lower than previously reported. However, our findings emphasize that Long COVID—rather than prior COVID-19 experience alone—is significant associated and with elevated levels of burnout and mental health challenges observed among healthcare workers. These results emphasize the continued impact of Long COVID poses for healthcare workers, even as the acute phase of the pandemic recedes. Our findings highlight the need for further research to fully understand the long-term consequences of Long COVID. Additionally, targeted interventions are needed to reduce burnout, enhance resilience, and improve psychological wellbeing of healthcare workers, who are essential to the healthcare system and play a vital role in safeguarding public health.

## Data Availability

The raw de-identified dataset supporting the conclusions of this article can be made available by the authors, without undue reservation, pending a data use agreement and IRB approval. Requests to access the datasets should be directed to charlesnika-evans@northwestern.edu.
