# Supplementary material for: Long COVID and its associations with burnout, anxiety, and depression among U. S. healthcare workers in the United States
Source: Front Public Health. 2025 Jul 9;13:1582872. doi: 10.3389/fpubh.2025.1582872 (PMC12283715; doi:10.3389/fpubh.2025.1582872)
Supplement: Supplementary file 1 [file Supplementary_file_1.docx]

**Supplementary Figure 1:** **Flowchart of Study Population Selection**

**
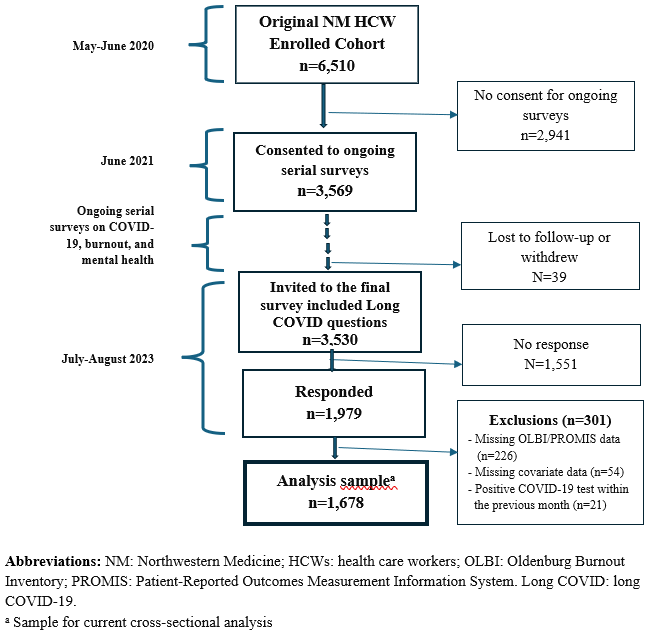
**

**Supplementary Figure 2: Adjusted**^a^ **Mean OLBI, PROMIS Depression, and Anxiety Scores by Long COVID Status**

Abbreviations: COVID+/LC+: had COVID-19 with Long COVID; COVID+/LC- : had COVID-19 without Long COVID; COVID+/LC? : had COVID-19 but unsure about Long COVID; COVID-/LC- : never had COVID-19

OLBI: Oldenburg Burnout Inventory; PROMIS: Patient-Reported Outcomes Measurement Information System.

^a^Models adjusted for age, sex, occupational status, BMI, number of comorbidities and number of vaccines received.

Y-axis omitted values from 0-30 scores.

**Supplementary Table 1: Multivariable^a^ Linear Regression Analysis of the Association between Long COVID and Burnout, Depression, and Anxiety—**excluding 209 participants who responded “unsure” to questions regarding their COVID-19 and Long COVID status (N=1,469)

| **Long COVID Status Comparisons** | **OLBI**  **β (SE), p-value** | **PROMIS Depression**  **β (SE), p-value** | **PROMIS Anxiety**  **β (SE), p-value** |
| --- | --- | --- | --- |
| COVID+/LC+ **vs** COVID+/LC- | **2.16 (0.77), 0.005** | **2.03 (0.77), 0.009** | **2.71 (0.77, 0.0004)** |
| COVID+/LC+ **vs** COVID-/LC- | **1.73 (0.82), 0.035** | 1.55 (0.82), 0.060 | **2.46 (0.82), 0.003** |
| COVID+/LC- **vs** COVID-/LC- | -0.43 (0.42), 0.308 | -0.47 (0.42), 0.253 | -0.26 (0.42), 0.540 |

Abbreviations: COVID+/LC+: had COVID-19 with Long COVID; COVID+/LC- : had COVID-19 without Long COVID; COVID+/LC? : had COVID-19 but unsure about Long COVID; COVID-/LC- : never had COVID-19

OLBI: Oldenburg Burnout Inventory; PROMIS: Patient-Reported Outcomes Measurement Information. System

^a^Models were adjusted for age, sex, occupational status, BMI, number of comorbidities and number of vaccines received.

**Supplementary Table 2: Adjusted^a^ Odds Ratios and 95% CI of Burnout, Depression, and Anxiety by Long COVID Status (N=1,678)**

| **Long COVID Status Comparisons** | **Burnout ^b^** | | | **PROMIS Depression^c^** | | | **PROMIS Anxiety^c^** | | |
| --- | --- | --- | --- | --- | --- | --- | --- | --- | --- |
|  | **OR** | **(95% CI)** | | **OR** | **(95% CI)** | | **OR** | **(95% CI)** | |
| **COVID+/LC+ vs COVID+/LC-** | **1.69** | **(1.08,** | **2.65)** | **1.65** | **(1.03,** | **2.66)** | **1.78** | **(1.13,** | **2.79)** |
| **COVID+/LC? vs COVID+/LC-** | 1.32 | (0.89, | 1.95) | **1.73** | **(1.15,** | **2.62)** | **1.63** | **(1.10,** | **2.41)** |
| **COVID+/LC+ vs COVID+/LC?** | 1.28 | (0.73, | 2.26) | 0.84 | (0.47, | 1.51) | 1.09 | (0.62, | 1.93) |
| **COVID+/LC+ vs COVID-/LC-** | 1.46 | (0.91, | 2.37) | 1.48 | (0.89, | 2.47) | **1.66** | **(1.02,** | **2.69)** |
| **COVID+/LC- vs COVID-/LC-** | 0.87 | (0.68, | 1.10) | 0.90 | (0.68, | 1.18) | 0.93 | (0.73, | 1.20) |
| **COVID+/LC? vs COVID-/LC-** | 1.14 | (0.75, | 1.75) | **1.77** | **(1.13,** | **2.75)** | 1.52 | (0.99, | 2.33) |

Abbreviations: OR: odds ratio; CI: confidence interval; PROMIS: Patient-Reported Outcomes Measurement Information System; COVID+/LC+: had COVID-19 with Long COVID; COVID+/LC- : had COVID-19 without Long COVID; COVID+/LC? : had COVID-19 but unsure about Long COVID; COVID-/LC- : never had COVID-19

^a^Models adjusted for age, sex, occupational status, BMI, number of comorbidities and number of vaccines received

^b^Burnout defined by average disengagement score ≥2.1 or average exhaustion score ≥ 2.25.

^c^Depression and anxiety defined as PROMIS T-scores ≥55

**Supplementary Table 3: Comparison of responders with non-responders to survey with Long COVID questions** (N=3,530)

| **Characteristic** | **Responders**  **N=1,979 (56.1%)** | **Non-responders**  **N=1,551 (43.9%)** | **P-value**^a^ |
| --- | --- | --- | --- |
| Age (mean, SD) | 45.8 (11.7) | 40.5 (11.2) | <0.0001 |
| Sex  Female (N/%) | 1612 (81.9%) | 1240 (81.1%) | 0.513 |
| Race/Ethnic Group (N/%)  Hispanic  Asian  Non-Hispanic White  Other | 114 (5.8%)  166 (8.4%)  1618 (81.8%)  81 (4.1%) | 113 (7.3%)  176 (11.4%)  1180 (76.1%)  82 (5.3%) | 0.0006 |
| Occupation (N/%)  Administration  Nurses  Physician  Other | 266 (13.5%)  575 (29.2%)  362 (18.4%)  766 (38.9%) | 180 (11.6%)  496 (32.4%)  316 (20.6%)  539 (35.2%) | 0.016 |
| Number of Comorbidities (mean, SD) | 0.5 (0.8) | 0.3 (0.6) | <0.0001 |

^a^P-value for overall group comparisons based on T-tests or Chi-square tests.
